# CD44 Promotes Breast Cancer Metastasis through AKT-Mediated Downregulation of Nuclear FOXA2

**DOI:** 10.3390/biomedicines10102488

**Published:** 2022-10-05

**Authors:** Anupama Vadhan, Ming-Feng Hou, Priya Vijayaraghavan, Yi-Chia Wu, Stephen Chu-Sung Hu, Yun-Ming Wang, Tian-Lu Cheng, Yen-Yun Wang, Shyng-Shiou F. Yuan

**Affiliations:** 1 Graduate Institute of Medicine, College of Medicine, Kaohsiung Medical University, Kaohsiung 807, Taiwan; 2 Division of Breast Oncology and Surgery, Department of Surgery, Kaohsiung Medical University Hospital, Kaohsiung 807, Taiwan; 3 Department of Biomedical Science and Environmental Biology, College of Life Science, Kaohsiung Medical University, Kaohsiung 807, Taiwan; 4 Division of Plastic Surgery, Department of Surgery, Kaohsiung Medical University Hospital, Kaohsiung 807, Taiwan; 5 School of Medicine, College of Medicine, Kaohsiung Medical University, Kaohsiung 807, Taiwan; 6 Department of Dermatology, College of Medicine, Kaohsiung Medical University, Kaohsiung 807, Taiwan; 7 Department of Dermatology, Kaohsiung Medical University Hospital, Kaohsiung 807, Taiwan; 8 Department of Biological Science and Technology, Institute of Molecular Medicine and Bioengineering, Center for Intelligent Drug Systems and Smart Bio-devices (IDS2B), National Yang Ming Chiao Tung University, 75 Bo-Ai Street, Hsinchu 300, Taiwan; 9 Department of Biomedical Science and Environmental Biology, Center for Cancer Research, Kaohsiung Medical University, Kaohsiung 807, Taiwan; 10 School of Dentistry, College of Dental Medicine, Kaohsiung Medical University, Kaohsiung 807, Taiwan; 11 Department of Biomedical and Environmental Biology, Kaohsiung Medical University, Kaohsiung 807, Taiwan; 12 Drug Development and Value Creation Research Center, Kaohsiung Medical University, Kaohsiung 807, Taiwan; 13 Department of Medical Research, Kaohsiung Medical University Hospital, Kaohsiung 807, Taiwan; 14 Translational Research Center, Kaohsiung Medical University Hospital, Kaohsiung 807, Taiwan; 15 Department of Obstetrics and Gynecology, Kaohsiung Medical University Hospital, Kaohsiung 807, Taiwan

**Keywords:** breast cancer, CD44, FOXA2, AKT, metastasis

## Abstract

The primary cause of breast cancer mortality is the metastatic invasion of cancerous stem cells (CSC). Cluster of differentiation 44 (CD44) is a well-known CSC marker in various cancers, as well as a key role player in metastasis and relapse of breast cancer. CD44 is a cell-membrane embedded protein, and it interacts with different proteins to regulate cancer cell behavior. Transcription factor forkhead box protein A2 (FOXA2) acts as an important regulator in multiple cancers, including breast cancer. However, the biological significance of CD44-FOXA2 association in breast cancer metastasis remains unclear. Herein, we observed that CD44 expression was higher in metastatic lymph nodes compared to primary tumors using a flow cytometric analysis. CD44 overexpression in breast cancer cell lines significantly promoted cell migration and invasion abilities, whereas the opposite effects occurred upon the knockdown of CD44. The stem cell array analysis revealed that FOXA2 expression was upregulated in CD44 knockdown cells. However, the knockdown of FOXA2 in CD44 knockdown cells reversed the effects on cell migration and invasion. Furthermore, we found that CD44 mediated FOXA2 localization in breast cancer cells through the AKT pathway. Moreover, the immunofluorescence assay demonstrated that AKT inhibitor wortmannin and AKT activator SC79 treatment in breast cancer cells impacted FOXA2 localization. Collectively, this study highlights that CD44 promotes breast cancer metastasis by downregulating nuclear FOXA2.

## 1. Introduction

Breast cancer is the most common malignancy affecting women worldwide. More than 90% of breast cancer patients succumb due to cancer metastasis to different organs such as bone, lung, brain, and liver [1,2]. Tumor heterogeneity in breast carcinoma refers to the presence of heterogeneous cell populations among different patients (inter-tumor heterogeneity) or within the same patient (intratumor heterogeneity), which leads to explicit manifestations of the disease [3]. Despite the tremendous advances in the knowledge of breast cancer heterogeneity, there exist several challenges to improve breast cancer diagnosis, treatment, and prognosis [3].

CD44 is a complex transmembrane adhesion glycoprotein that exists in various molecular forms, including the standard and CD44 variant isoforms [4]. CD44 is inherently associated with the key constituents of the extracellular matrix (ECM) and hyaluronic acid (HA) [4]. CD44 interacts with various other cell surface receptors to promote the activation of different signaling pathways such as Rho GTPases, Ras-MAPK, and PI3K/AKT, which regulate cell migration, survival, invasion, and epithelial–mesenchymal transition (EMT) [5,6,7]. CD44 has also been found to play a role in cellular signaling and cell–cell communication through complex formation between extracellular components and intracellular cytoskeletal elements [8]. Furthermore, CD44 has been implicated in sensing changes in ECM and cellular microenvironment and influences various cell behaviors, including cell survival, growth, differentiation, and motility [9].

CD44 is also a well-known surface biomarker of CSCs, and any anomalous expression or dysregulation of CD44 may indicate tumorigenesis and metastasis in multiple cancers such as colon [10,11,12], bladder [13], gastric [14], lung [15,16], and breast cancers [17,18,19,20,21]. It has been reported that CD44 expression correlates with tumor grade and tumor recurrence in breast cancer patients and also promotes metastasis [21]. In a meta-analysis study, it has been reported that CD44 is associated with EMT and the cancer stem cell gene profile [22]. Studies on tetracycline-induced CD44 expression have also been reported in noninvasive luminal MCF7 cell lines [23]. Furthermore, another study has demonstrated the role of CD44 in promoting breast cancer invasion and tumor metastasis to liver in vivo [12].

Forkhead box protein A2 (FOXA2), also known as hepatocyte nuclear factor 3-beta (HNF-3B), is a pioneer transcription factor that belongs to the forkhead/winged-helix family of transcription factors [24]. Various members of the FOX transcription factor family are widely distributed in eukaryotes [25]. These transcription factors contain a forkhead domain (also known as the winged-helix domain) flanked by the sequences required for nuclear localization [26]. FOXA2 plays a significant role in the formation of node, notochord, nervous system, and endoderm-derived structures [27]. Additionally, FOXA2 is a key regulator in embryonic development and the normal functioning of various cells and tissues [24].

Several studies have confirmed the role of FOXA2 as a tumor suppressor gene or oncogene in different cancers by activating or downregulating different pathways and proteins [25,26,27,28,29,30,31,32,33]. In lung cancer, FOXA2 has been reported to be downregulated [28] and inhibits lung cancer cell proliferation and metastasis [27,29]. Additionally, FOXA2 is downregulated by miR-590-3p in ovarian cancer, which promotes cancer growth and metastasis [30]. Similarly, FOXA2 has been reported to be a tumor suppressor gene in various cancers and is a target of oncogenes, such as in pancreatic cancer [31], liver cancer [32], oral cancer [33,34], and cervical cancer [35]. On the other hand, FOXA2 has been reported to promote EMT in colon cancer and prostate cancer [36,37]. Nonetheless, targeting FOXA2 by various microRNAs has been shown to promote cancer metastasis and proliferation [38,39]. In breast cancer, FOXA2 is known to attenuate EMT by regulating E-cadherin and ZEB2 expression [24]. Likewise, a previous study suggests that the interaction between FOXA2 and FOXP2 could inhibit EMT by activating E-cadherin and PHF2 transcription genes [23]. Additionally, the overexpression of FOXA2 combined with the downregulation of PGC-1β has been recently reported to inhibit breast cancer proliferation and migration and induce apoptosis [38].

In this study, we analyzed breast cancer patient samples to evaluate CD44 expression using flow cytometry. It was found that CD44 expression was higher in metastatic lymph nodes compared to primary tumors. Moreover, the overexpression of CD44 promoted breast cancer migration and invasion. On the other hand, the knockdown of CD44 suppressed the migration and invasion of breast cancer cells. Additionally, a stem cell microarray analysis showed that FOXA2 expression was upregulated in CD44 knockdown cells. The molecular mechanistic studies revealed that CD44 plays a pivotal role in controlling FOXA2 localization to promote cancer metastasis via the AKT signaling pathway.

## 2. Materials and Methods

### 2.1. Human Specimens

Primary tumors and lymph node specimens from female breast cancer patients were obtained following surgical treatment at Kaohsiung Medical University Hospital under an Institutional Review Board-approved protocol (KMUH-IRB-20130346).

### 2.2. Cell Culture

Human breast carcinoma cells (luminal types: MCF7, T47D, and ZR75; basal types: MDA-MB-231 and HS578T) were obtained from the Bioresource Collection and Research Center (BCRC; https://www.bcrc.firdi.org.tw/12092013 (accessed on 30 May 2022)). Cells were maintained in DMEM (Gibco) with 5% CO_2_ at 37 °C in a humidified incubator. All cell culture media were supplemented with 10% FBS (Biological Industries) and 1% PSA (penicillin G/streptomycin/amphotericin B; Biological Industries). Kinase inhibitor wortmannin (20 μM; Sigma) and kinase activator SC79 (5 μM; Sigma) were used to investigate the Akt signaling pathway. Three chemotherapy drugs, fluorouracil (5FU) (Sigma), paclitaxel (Sigma), and doxorubicin (Sigma), were used to study chemoresistance in CD44-overexpressing and knockdown cells.

### 2.3. Transwell Migration and Invasion Assays

Cell migration and invasion assays were performed as described in our previous studies [40,41]. Briefly, breast cancer cells resuspended in serum-free cell culture medium were transferred onto Corning Costar Transwell inserts (3 × 10^4^ cells/8-μm pore size insert; Merck, Kenilworth, NJ, USA) in 24-well plates prefilled with complete cell culture medium in the bottom wells. Inserts containing Corning Matrigel coating (Merck, Kenilworth, NJ, USA) were used for the cell invasion assay, while inserts without Matrigel coating were used for the cell migration assay. After 24-h incubation, cells remaining on the upper side of the inserts were removed by cotton swabs, while those appearing on the underside of the inserts were fixed and stained with crystal violet. The images were captured by a light microscope, and the results were analyzed by ImageJ software (https://imagej.nih.gov/ij/08112014 (accessed on 20 May 2022). Three replicates were used for all migration and invasion assays.

### 2.4. Western Blo

The total protein lysates were extracted in RIPA lysis buffer (150 mM NaCl, 1% IGEPAL CA-630, 0.5% sodium deoxycholate, 0.1% SDS, and 50 mM Tris, pH8.0) [40]. The protein concentration was measured by the Bio-Rad protein assay kit (BIO-RAD Laboratories, Heracles, CA, USA). Equal amounts of total protein were fractionated by SDS-PAGE electrophoresis and transferred to a nitrocellulose membrane (Millipore, Burlington, MA, USA). A pre-stained protein ladder (Thermo Fisher Scientific, New York, NY, USA) was used as the molecular weight standard. After incubation with 5% nonfat milk, the membranes were incubated with designated primary antibodies at 4 °C overnight. Immunoreactive proteins were detected after incubation with horseradish peroxidase-conjugated secondary antibody (Thermo Fisher Scientific, Waltham, MA, USA) for 1 h at room temperature. The immunoblots were visualized by using enhanced chemiluminescence Western Lightning Plus-ECL (PerkinElmer, Waltham, MA, USA), and the images were captured by the ChemiDoc XRS+ imaging system (BIO-RAD Laboratories, Heracles, CA, USA) and quantified by Image Lab software (BIO-RAD Laboratories, Heracles, CA, USA). Antibodies against β-actin (1:5000, A5441) were purchased from Sigma Aldrich (Burlington, MA, USA). Antibodies against E-cadherin (GTX52341, 1:2000), AKT (GTX110613, 1:2000), Lamin-A/C (GTX101127, 1:5000), GAPDH (GTX 100118, 1:10,000), and CD44 (GTX102111, 1:3000) were purchased from GenTax (Taiwan). Antibodies against FOXA2 (ARG63339, 1:1000) and p-AKT (Cell signaling #3787, 1:1000) were purchased from Arigo (Glasgow, UK) and Cell Signaling (Danvers, MA, USA), respectively. HRP-conjugated secondary antibodies were purchased from Thermo Fisher Scientific (Waltham, MA, USA). Densitometry results were obtained from three independent Western blots.

### 2.5. Immunohistochemistry

Immunohistochemical staining for CD44 was performed with the fully automated Bond-Max system according to the manufacturer’s instructions (Leica Microsystems, Wetzlar, Germany). For quantification, the protein expression levels were scored using the method of histochemical score (H-score), as described in our previous studies [40,41,42,43]. The H-score was calculated as the product of the percentage (0–100%) of stained cells and intensity of staining (0–3). The discriminatory threshold was set at H-score = 200 and existing samples were reread and classified as low (H-score < 200) or high (≥200) CD44 expression. Two independent specialists made the determination of staining for each sample simultaneously and separately under the same circumstances.

### 2.6. Flow Cytometry

Primary tumors and paired metastatic lymph nodes were collected from breast cancer patients and dissociated by the gentleMACS Dissociator (Miltenyi Biotec, Bergisch Gladbach, Germany). To remove dead cells, the Debris Removal Solution (130-109-398) was used as per the manufacturer’s instructions. Anti-CD45 magnetic beads (11153D, Thermo Fisher Scientific, Invitrogen, Waltham, MA, USA) were added to 2 × 10^7^ cells and incubated at 4 °C for 30 min to remove the immune cells. Finally, the cells were placed on a magnetic platform for 10 min before being extracted (1 × 10^5^ cells) for the flow cytometry analysis. Fluorochrome-conjugated antibodies against CD44 (11-0441-82, Thermo Fisher Scientific, eBioscience, Waltham, MA, USA) conjugated with PE-Cy7 (BD Biosciences, San Jose, CA, USA) and CD24 (45-0242-82, Thermo Fisher Scientific, eBioscience manufacturer, Waltham, MA, USA) were used to label the cells before the flow cytometry analysis. The immunostained cells were detected using a BD FACSCalibur flow cytometer (BD Biosciences, San Jose, CA, USA) and analyzed by FlowJo ver. 7.6.1 software (BD Biosciences, San Jose, CA, USA).

### 2.7. Tumorsphere Formation Assay

Breast cancer cells were seeded onto Corning Costar Ultra-Low Attachment 96-well plates (Merck, Kenilworth, NJ, USA) at a density of 1 × 10^3^ cells/well with phenol red-free DMEM (Thermo Fisher Scientific, New York, USA) containing 20 ng/mL EGF (PeproTech, Rehovot, Israel), 20 ng/mL basic FGF (PeproTech, Rehovot, Israel), 10 μg/mL insulin (Merck, Kenilworth, NJ, USA), and 1× B27 (Thermo Fisher Scientific, New York, NY, USA). The cells were cultured under normal cell culture conditions for 7 days prior to the assessment for tumorsphere formation. The plates were imaged under a light microscope and analyzed by ImageJ software for tumorspheres with a diameter over 50 μm.

### 2.8. Human Cancer Stem Cell Array

Total RNA was extracted using the TRIzol Reagent (Thermo Fisher Scientific, New York, NY, USA) according to the manufacturer’s instructions. An aliquot of RNA (2 μg/sample) was treated with DNase (Merck, Kenilworth, NJ, USA) and transcribed into cDNA using the RT^2^ First Strand Kit (Qiagen, MD, USA), followed by the procedures to screen for 84 cancer stem cell-associated genes with the human cancer stem cell RT^2^ Profiler PCR Array (Cat. No. 330231 PAHS-176ZA, Qiagen), as described previously [44].

### 2.9. Gene Knockdown and Overexpression

To knockdown *CD44* in breast cancer cells MDA-MB-231 and MCF7, a lentivirus carrying a pLKO.1_puro lentiviral vector that expresses double-stranded shRNA oligonucleotides targeting the sequences of human CD44 (2 clones) was used (Clone 1: IDTRCN0000296191 and Clone 2: ID TRCN0000308110 (National RNAi Core Facility, Academia Sinica, Taipei, Taiwan) (Table S1)). ShRNA used in this study targets the canonical CD44 (CD44s) standard isoform. Another pLKO.1_puro lentiviral vector expressing shRNA targeting firefly luciferase, which is not related to the human genome sequence, was used as a negative control (National RNAi Core Facility, Academia Sinica, Taipei, Taiwan). FOXA2 shRNA was also purchased from the National RNAi Core Facility, Academia Sinica, Taiwan.

To overexpress *CD44* in breast cancer cells ZR75 and MCF7, a ready-to-use lentivirus particle with the pReceiver Lv105 lentiviral vector, which expresses the human *CD44* gene, was purchased from Genecopoeia (Rockville, MD, USA). For the negative control, lentivirus particles that carry an empty pReceiver Lv105 lentiviral vector were used (Genecopoeia, Rockville, MD, USA). To knockdown or overexpress *CD44* in breast cancer cells, MDA-MB-231, MCF7, and ZR75 were seeded on 6-well plates at a density of 2 × 10^5^ cells/well 24 h prior to the lentiviral transduction. Lentiviral virus solution (MOI = 5) was added to cells in the culture medium containing 8 μg/mL polybrene. Forty-eight hours after infection, the virus-containing medium was replaced with 2 μg/mL puromycin-containing medium and incubated for 48–72 h (duration dependent on noninfected cells that were used as the negative control) to select knockdown cells. Surviving cells were maintained with 1 μg/mL puromycin for 1 to 2 weeks (based on cell proliferation) until further experiments.

Similar steps were followed to knockdown *FOXA2* in CD44 knockdown cells. A large amount (400 µg/mL) of neomycin (G418) was used for selection of the *FOXA2* knockdown cell population in CD44 knockdown cells.

### 2.10. Statistics

Data from three independent experiments were presented as the mean ± SD. Individual statistical tests are mentioned in the figure legends, with statistical significance established at *p* < 0.05. All statistical analyses were conducted using Prism 8.0 software (GraphPad, La Jolla, CA, USA).

## 3. Results

### 3.1. CD44 Expression Was Higher in Metastatic Lymph Nodes and CD44 Knockdown Reduced Migration and Invasion Abilities of Breast Cancer Cells

**Figure 1:**
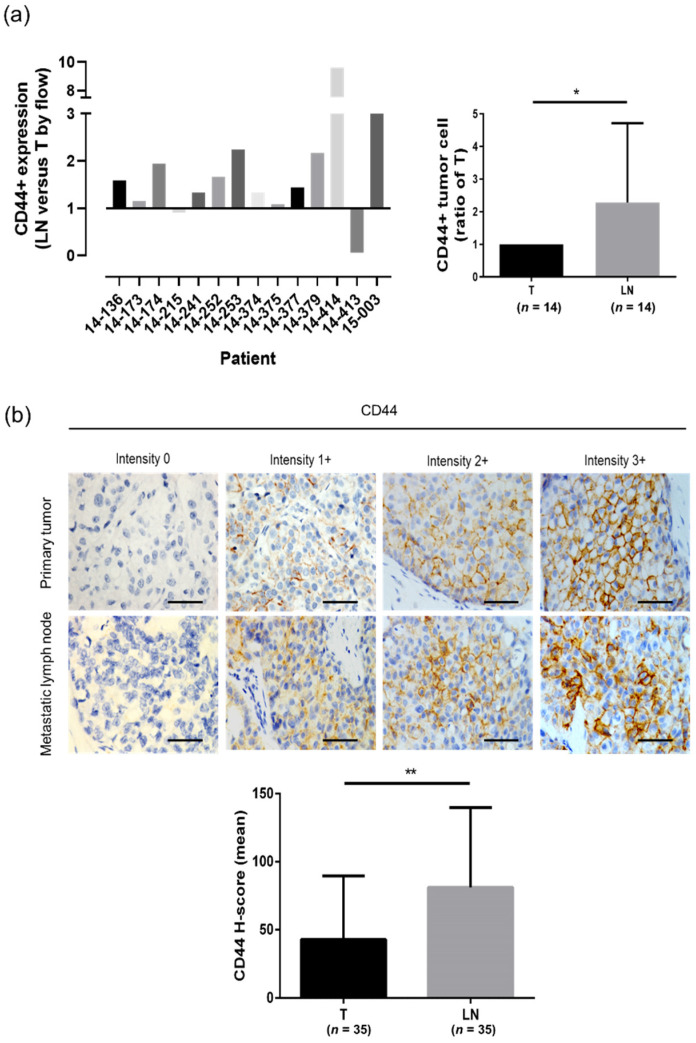

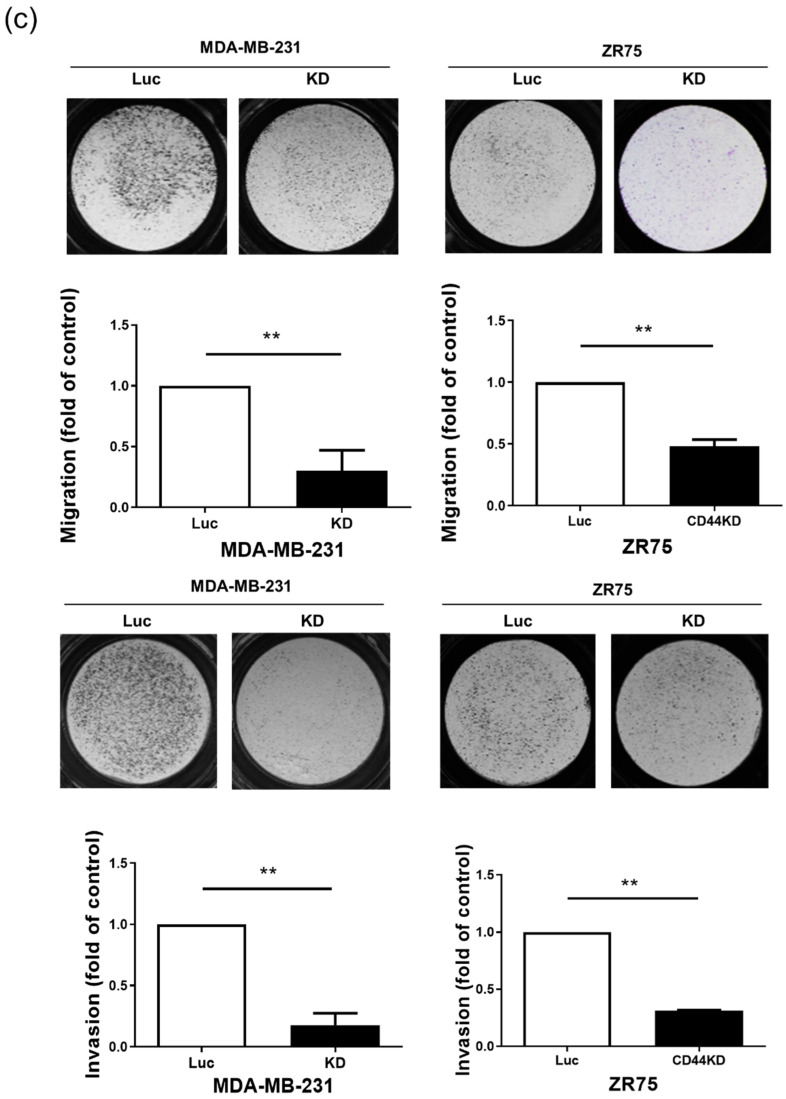

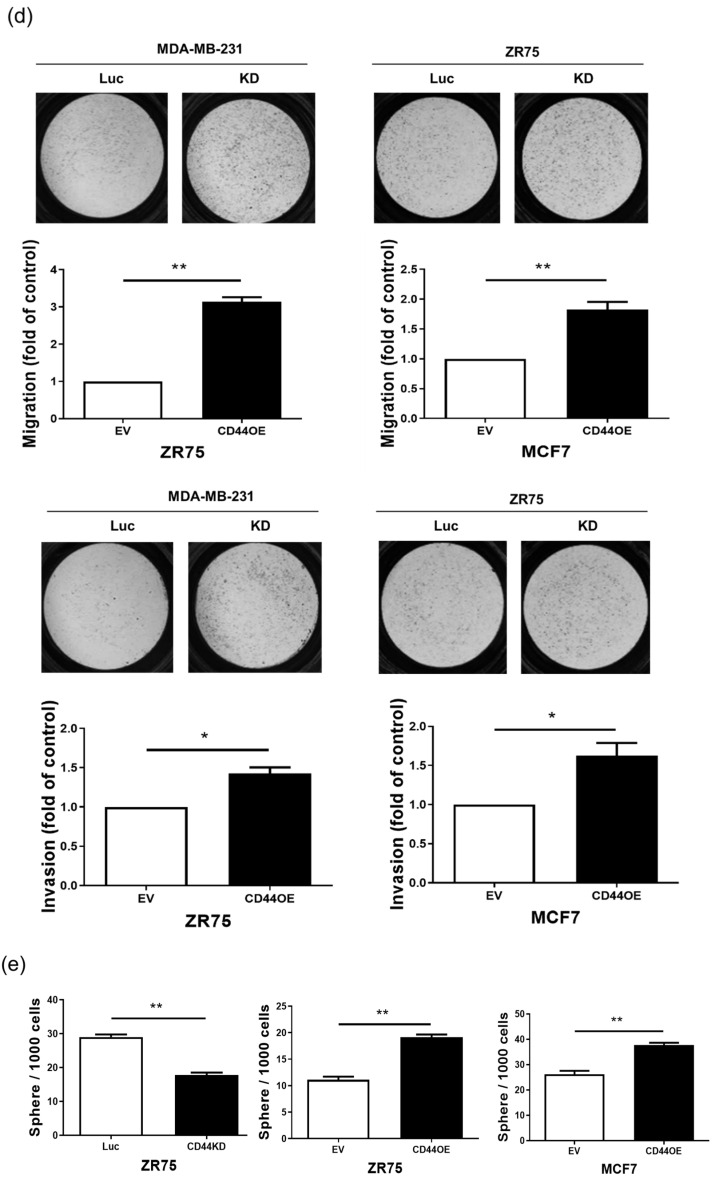
CD44 expression was higher in metastatic lymph nodes, and CD44 knockdown reduced the migration and invasion abilities of breast cancer cells: (**a**) Flow cytometric analysis of CD44 expression in primary breast tumors (T) and paired metastatic lymph nodes (LN) in 14 breast cancer patients’ samples. (**b**) The expression of CD44 in breast cancer tissues, as determined by immunohistochemistry, was assessed by the H-score. The discriminatory threshold was set at H-score = 200, and existing samples were reread and classed as low (H-score < 200) or high (≥200) CD44 expression. (left) Bar figure showing the quantification results for CD44 expression in primary tumors (T) and lymph node metastases (LN) was assessed by immunohistochemistry in 35 breast cancer patients’ samples. Scale bar = 100µm. (**c**) Cell migration and invasion abilities in control and CD44 knockdown MDA-MB-231 and ZR75 cells (Luc: luciferase, KD: knockdown). (**d**) Cell migration and invasion abilities in control and CD44-overexpressing ZR75 and MCF7 cells (EV: empty vector, OE: overexpression). (**e**) Mammosphere formation ability of control, CD44 knockdown ZR75, and CD44 overexpressed ZR75 and MCF7 cells. The data are presented as mean ± SD. * Indicates *p* < 0.05, and ** indicates *p* < 0.01.

In order to analyze the CD44-positive cell population in primary tumors and lymph nodes, tissue samples were collected from 14 breast cancer patients. The results from the flow cytometric analysis showed that there was a markedly higher percentage of CD44-expressing cells in metastatic lymph nodes compared to primary tumors (Figure 1a; Figure S1a,b). Additionally, immunohistochemical staining showed that metastatic lymph nodes had a higher expression of CD44 compared to primary tumors (Figure 1b). Subsequently, the expression of CD44 was investigated in different breast cancer cell lines (Figure S1c). The results revealed that basal-type cancer cells had elevated levels of CD44 expression. In contrast, luminal cells showed a low expression of CD44. To evaluate the effects of CD44 on the phenotype of human breast cancer cells, CD44 expression was knocked down in MDA-MB-231 and ZR75 cells. First, we checked the knockdown efficiency of two clones and found that only clone 2 showed good knockdown efficiency (Figure S1d). Therefore, we used only clone 2 for further experiments. Conversely, we overexpressed CD44 in MCF7 and ZR75 cells. It can be seen that the migration and invasion abilities of cancer cells declined significantly in CD44 knocked down cells and vice versa (Figure 1c,d). Interestingly, CD44 knockdown and overexpression induced morphological changes in MDA-MB-231 cells that can be clearly observed in the optical microscopic images. ZR75 cells displayed a typical epithelial morphology (Figure S1e). On the other hand, CD44-overexpressed MCF7 and ZR75 cells became spindle-shaped (Figure S1e). Additionally, the cell viability of CD44 knocked down MDA-MB-231 cells was found to be decreased, while the cell viability of CD44 overexpressed MCF7 cells was increased (Figure S1f). Furthermore, cancer stemness was decreased in CD44 knocked down ZR75 cells and increased in CD44 overexpressed MCF7 and ZR75 cells (Figure 1e).

### 3.2. FOXA2 Was Upregulated in CD44 Knockdown Cells

**Figure 2:**
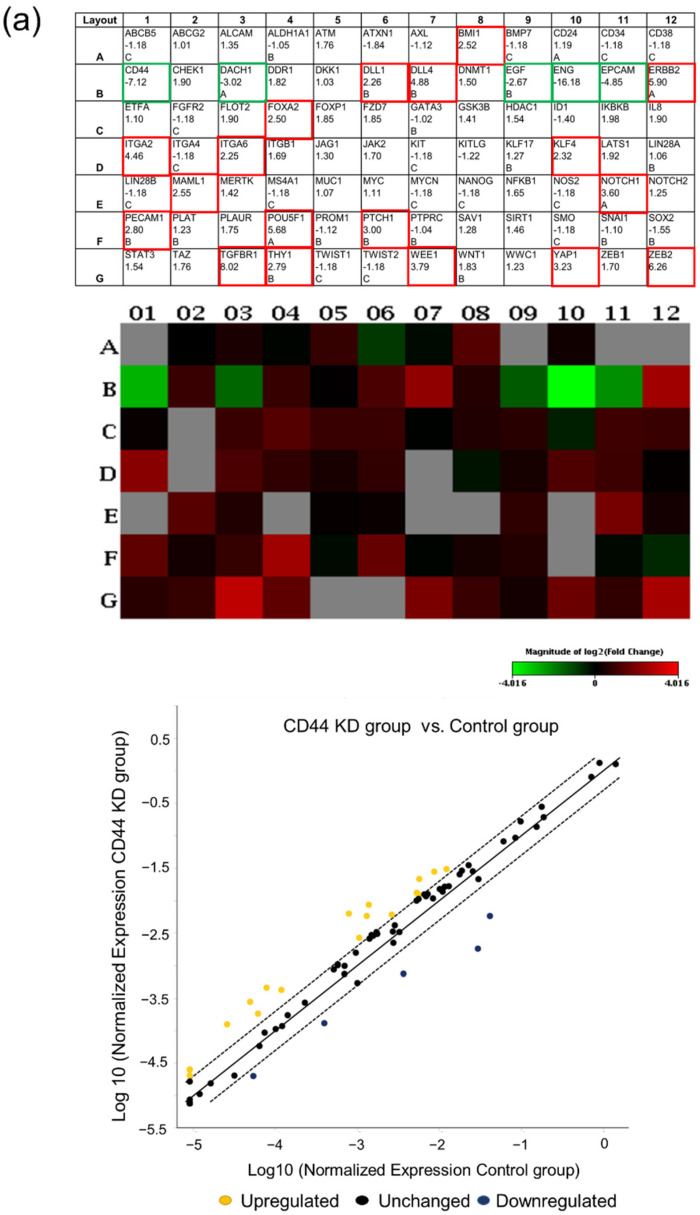

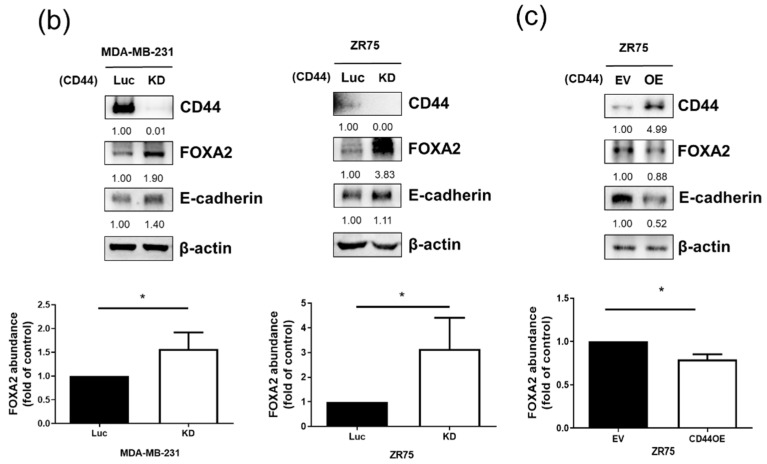
FOXA2 was upregulated in CD44 knockdown cells: (**a**) Human stem cell array table used in RT^2^ profiler PCR array experiments (top). Heat map showing the altered expression of stem cell array genes (middle); red, green, and black squares indicate upregulated, downregulated, and nonregulated genes, respectively. Scatter plot showing genes with >2-fold difference in mRNA expression (bottom) in CD44 knockdown MDA-MB-231 cells, as identified by using the human cancer stem cell array. (**b**) Immunoblotting of CD44, FOXA2, and E-cadherin in nontargeting control and CD44 knockdown cells (MDA-MB-231 and ZR75). Knockdown clone 2 was used to knockdown CD44 in MDA-MB-231 and ZR75 cells. (**c**) Immunoblotting of CD44, FOXA2, and E-cadherin in nontargeting control and CD44-overexpressed ZR75 cells. The data are presented as the mean ± SD. * Indicates *p* < 0.05.

To investigate the downstream event of CD44, a human cancer stem cell RT^2^ Profiler PCR Array was applied to MDA-MB-231 CD44 knockdown cells. We discovered that the mRNA levels of various genes were upregulated in CD44 knockdown cells, and we focused on FOXA2, which has been reported to inhibit epithelial to mesenchymal transition in breast cancer [24,25,45] (Figure 2a; Figure S2a). Next, we confirmed our stem cell array results by Western blot (Figure 2b). In previous studies, FOXA2 has been reported to promote E-cadherin expression [24,33]. Therefore, we also checked E-cadherin protein expression and found that it was increased in CD44 knockdown cells (Figure 2b) and decreased in CD44 overexpressed cells (Figure 2c). Additionally, we evaluated the mRNA level of FOXA2 in CD44 knocked down MDA-MB-231 cells and CD44-overexpressed MCF7 cells. The results showed that the FOXA2 mRNA level was markedly elevated in CD44 knockdown cells compared to CD44-overexpressed cells (Figure S2b,c).

### 3.3. Inhibition of FOXA2 in CD44 Knockdown Cells Reversed Cell Phenotype from Epithelial to Mesenchymal

**Figure 3:**
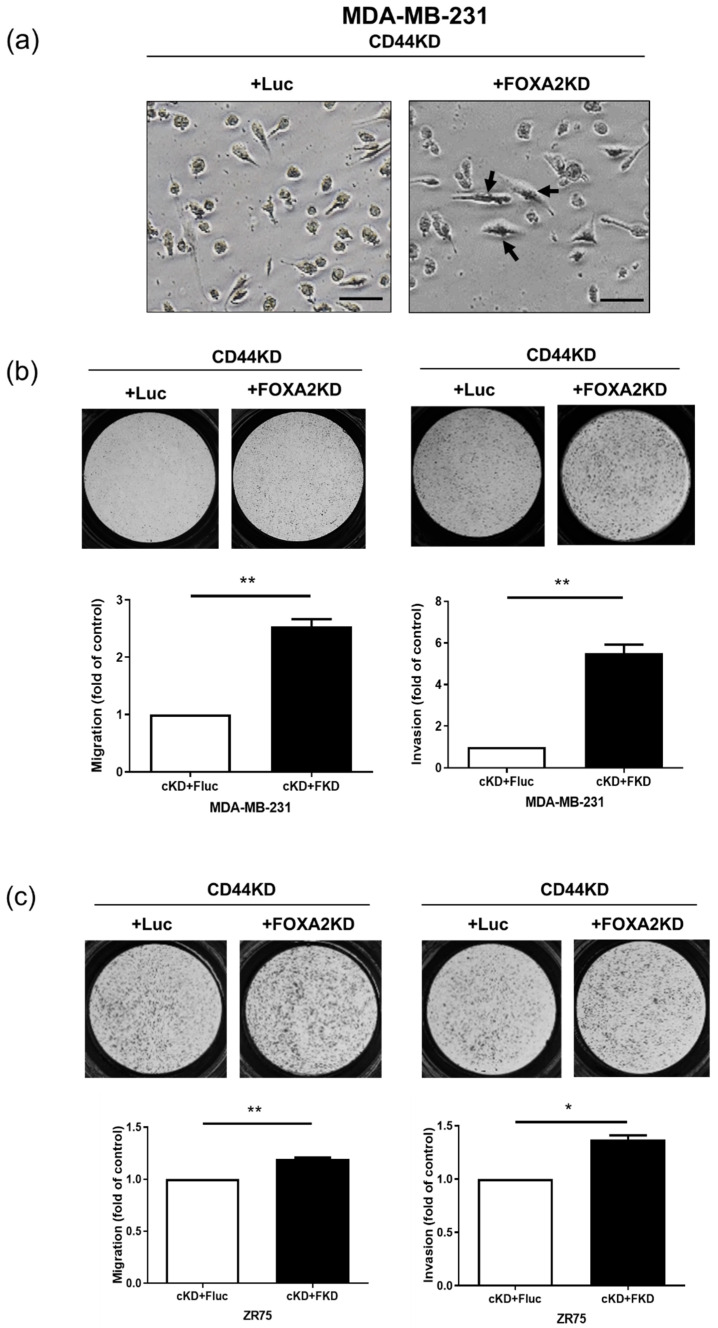
FOXA2 knockdown in CD44 knockdown cells reversed the epithelial phenotype to a mesenchymal phenotype: (**a**) Morphology of MDA-MB-231 cells with the double knockdown of CD44 and FOXA2. Scale bar = 50 µm. (**b**,**c**) Migration and invasion abilities of MDA-MB-231 and ZR75 cells with the double knockdown of CD44 and FOXA2. The data are presented as the mean ± SD. * Indicates *p* < 0.05, and ** indicates *p* < 0.01.

Furthermore, we knocked down FOXA2 in CD44 knockdown cells. Interestingly, the morphology of the double knockdown cells changed from a round shape to a spindle shape (Figure 3a). Additionally, FOXA2 inhibition reversed the phenotype of CD44 knockdown cells and increased the migration and invasion abilities of MDA-MB-231 and ZR75 cells with the double knockdown of CD44 and FOXA2 (Figure 3b,c). We checked the protein expression of mesenchymal markers snail, slug, twist, vimentin, and ZEB1 in MDA-MB-231 cells with CD44 knockdown and found that their expression was downregulated, except ZEB1 (Figure S3a), which is in agreement with previous reports that the expression of mesenchymal markers is upregulated as a result of CD44 overexpression. We also found that E-cadherin expression was downregulated in CD44 and FOXA2 double knockdown MDA-MB-231 cells (Figure S3b). Further study on the expression of mesenchymal markers snail, twist, and ZEB1 proteins in CD44 and FOXA2 double overexpressing cells (Figure S3c) showed that CD44 overexpression only upregulated the snail and twist expression but downregulated ZEB1 expression, while double overexpression (CD44 and FOXA2) reversed the twist expression but not the snail or ZEB1 expression (Figure S3c).

### 3.4. CD44 Leads to Multiple Drug Resistance in Breast Cancer Cells

**Figure 4:**
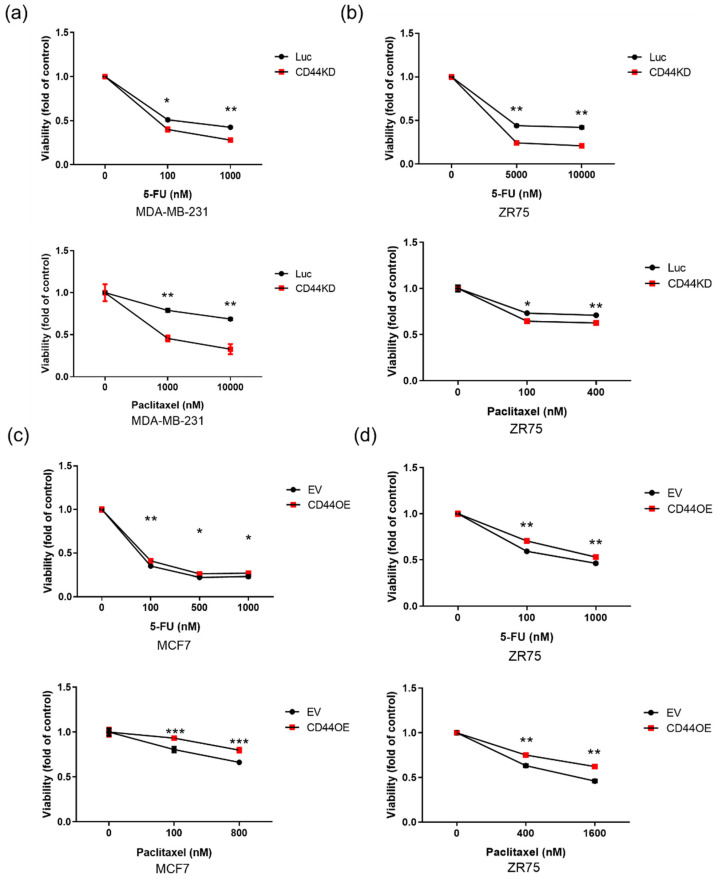
CD44 facilitates multiple drug resistance in breast cancer cells: (**a**,**b**) Effects of 5-FU and paclitaxel on CD44 knockdown MDA-MB-231 and ZR75 cells. (**c**,**d**) Effects of 5- FU and paclitaxel on CD44 overexpressed MCF7 and ZR75 cells. Two-way ANOVA was performed for the statistical analysis. The data are presented as the mean ± SD. * Indicates *p* < 0.05, ** indicates *p* < 0.01, and *** indicates *p* < 0.001.

Previously, it has been reported that CD44 is a cancer stem cell marker, and stem cells usually display drug resistance [18]. Hence, the role of CD44 in cancer drug resistance in CD44 knockdown MDA-MB-231 and ZR75 cells, along with CD44-overexpressed MCF7 and ZR75 cells, was evaluated by using chemotherapy drugs, including fluorouracil (5-FU), paclitaxel, and doxorubicin. The results demonstrated that the viability of cells decreased markedly in the CD44 knockdown group after treatment with different drugs (Figure 4a,b; Figure S4a), while CD44 overexpressed cells showed resistance to all three drugs (Figure 4c,d; Figure S4b).

### 3.5. FOXA2 Accumulates in the Nucleus in CD44^low^ Breast Cancer Cells

**Figure 5:**
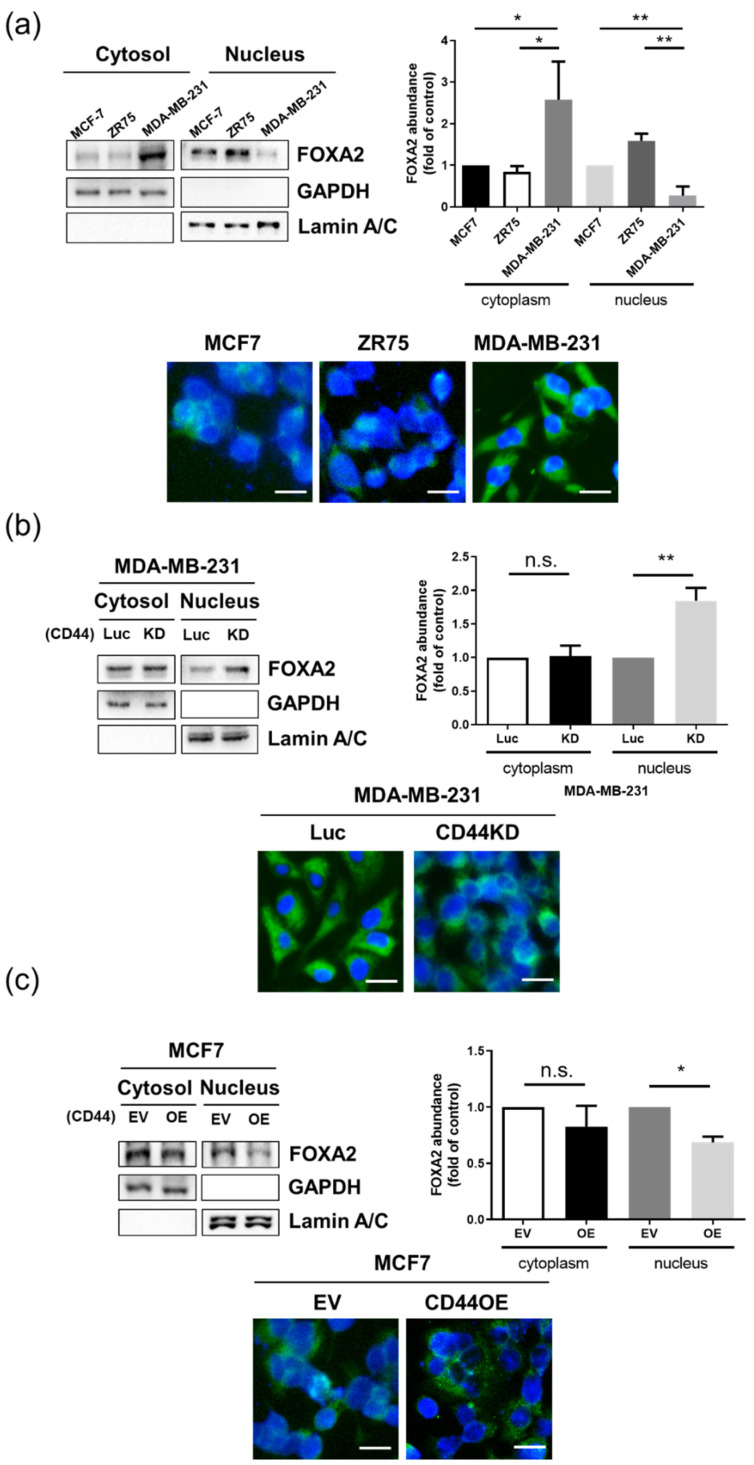
FOXA2 accumulates in the nucleus in CD44^low^ breast cancer cells: (**a**) Immunoblotting, quantification, and immunofluorescence staining of nuclear and cytoplasmic FOXA2 protein in MDA-MB-231, ZR75, and MCF7 cells. (**b**) Immunoblotting, quantification, and immunofluorescence staining of nuclear and cytoplasmic FOXA2 protein in CD44 knockdown MDA-MB-231 cells. (**c**) Immunoblotting, quantification, and immunofluorescence staining of nuclear and cytoplasmic FOXA2 protein in CD44-overexpressed MCF7 cells. n.s. stands for not significant. Scale bar = 50 µm. The data are presented as the mean ± SD. * Indicates *p* < 0.05, and ** indicates *p* < 0.01.

To further investigate how CD44 can affect FOXA2 expression, we evaluated FOXA2 localization, as FOXA2 is a transcription factor, and it acts in the nucleus. Using subcellular fractionation in three breast cancer cell lines, MDA-MB-231, MCF7, and ZR75, we found that, in mesenchymal breast cancer cell line MDA-MB-231, the cytosolic expression of FOXA2 was higher compared to the nucleus. On the other hand, epithelial breast cancer cells MCF7 and ZR75 showed high FOXA2 expression in the nucleus. We also confirmed that our results were consistent with the immunofluorescence staining for FOXA2 (Figure 5a). In addition, we investigated the localization of FOXA2 in CD44 knockdown and overexpressed cells by using subcellular fractionation and immunofluorescence staining. It was found that, in CD44 knockdown cells, the nuclear expression of FOXA2 was significantly increased (Figure 5b), while, in CD44-overexpressed MCF7 cells, the FOXA2 nuclear expression was decreased compared to the control group (Figure 5c). All these findings suggest that CD44 plays a role in regulating FOXA2 localization in breast cancer cells.

### 3.6. AKT Activation Results in Cytoplasmic Translocation of FOXA2

**Figure 6:**
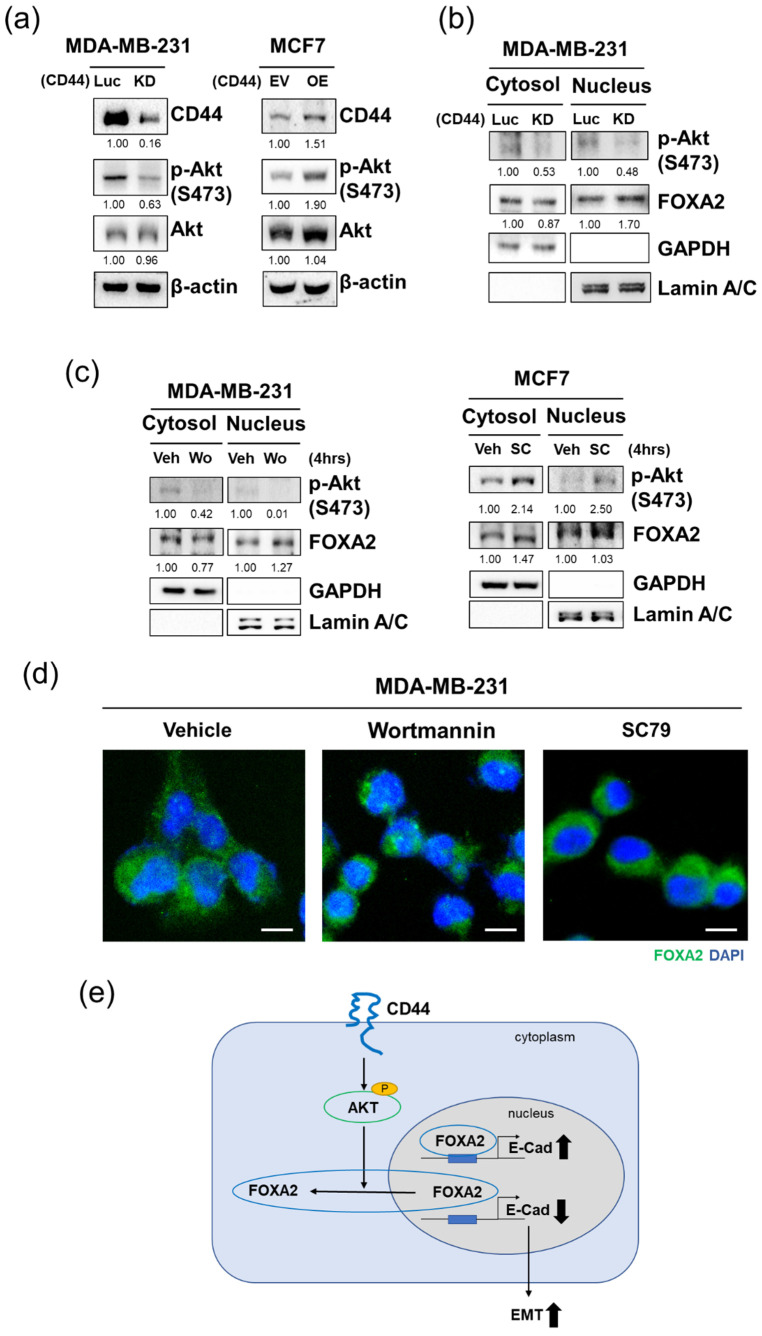
AKT activation results in the cytoplasmic translocation of FOXA2: (**a**) Immunoblotting for AKT and p-AKT protein expression in CD44 knockdown and overexpressed MDA-MB-231 and MCF7 cells, respectively. (**b**) Immunoblotting for the cytoplasmic and nuclear expression of p-AKT and FOXA2 in CD44 knockdown MDA-MB-231 cells. (**c**) Immunoblotting for the cytoplasmic and nuclear protein expression of p-AKT and FOXA2 in Veh (DMSO used as the vehicle), wortmannin (Wo), and SC79 (SC)-treated MDA-MB-231 and MCF7 cells, respectively. (**d**) Immunofluorescence staining for FOXA2 (green) in wortmannin (Wo) and SC79 (SC)-treated MDA-MB-231 cells. Scale bar = 50 µm. (**e**) Summary diagram for the signaling pathway.

Aiming to investigate the possible downstream effectors of CD44, which may regulate FOXA2 localization, we used the NetPhos 2.0 Server (http://www.cbs.dtu.dk/services/NetPhos/21122017 (accessed on 30 May 2022), HSLS, Pennsylvania, USA) to predict phosphorylation sites [46] in the FOXA2 protein, and then, the KinasePhos 2.0 Server was used to predict the kinase-specific site on the basis of an amino acid coupling pattern analysis [47]. The results indicated that AKT is the possible kinase through which CD44 regulates FOXA2 localization (Figure S5a). Based on this, AKT expression was analyzed by Western blotting, which showed that phosphorylated AKT expression was decreased in CD44 knockdown cells and increased in CD44 overexpressed cells (Figure 6a; Figure S5b). A previous study also found that CD44 facilitates signaling activation through the PI3K/AKT pathway [48]. Furthermore, subcellular fractionation in CD44 knockdown MDA-MB-231 cells revealed that p-AKT expression was decreased in both the cytoplasm and nucleus (Figure 6b). To confirm our finding that AKT indeed affects FOXA2 localization, we treated MDA-MB-231 cells with wortmannin, which is an AKT inhibitor. It was found that FOXA2 expression was increased in the nucleus and decreased in the cytoplasm (Figure 6c). In contrast, the AKT activator SC79 increased the cytosolic expression of FOXA2 in MCF7 cells in which FOXA2 expression is normally low in the cytoplasm (Figure 6c). Immunofluorescence staining was performed to further confirm the changes in FOXA2 localization after wortmannin and SC79 treatment (Figure 6d, Figure S5c). Taken together, these results suggest that CD44 regulates FOXA2 localization through AKT and promotes metastasis in breast cancer cells.

## 4. Discussion

It has been reported that CD44 promotes stemness and metastasis in various cancers, including breast cancer [15,19,21,49,50,51,52]. It is also well-documented that CD44 promotes cancer cell migration and invasion via mesenchymal markers [49,53,54,55]. This study has added new information to the growing body of evidence that CD44 plays an important role in breast cancer metastasis and multidrug resistance. For the first time, we have found that CD44 regulates FOXA2 localization through AKT to promote the metastatic ability of breast cancer cells.

In the current study, we found that metastatic lymph nodes showed higher expression of CD44 compared to primary tumors in tissue samples collected from breast cancer patients. Additionally, the migration and invasion abilities were decreased in CD44 knocked down breast cancer cells and increased in CD44-overexpressed cells. We also found morphological changes in breast cancer cells after the knockdown and overexpression of CD44. The CD44 knockdown cells acquired an epithelial phenotype, whereas CD44 overexpressed cells showed a mesenchymal phenotype. Our findings were consistent with the previous studies on breast cancer migration and metastasis. Previous studies reported that CD44 promotes the distant metastasis of breast cancer cells in vivo [21]. Additionally, CD44 upregulation in breast cancer has been correlated with a higher tumor grade [56]. In a meta-analysis, elevated CD44 expression has been reported in the basal subtype of breast cancer and was associated with the EMT and cancer stem cell signature [22]. CD44 can also modulate breast cancer metabolism under hypoxic conditions and promote EMT [57].

From the stem cell array analysis, we found that FOXA2 expression was upregulated in CD44 knockdown cells. FOXA2 has been reported as a tumor suppressor in different cancers. In hepatocellular carcinoma, FOXA2 suppresses metastasis partially through matrix metalloproteinase-9 inhibition [58]. In gastric cancer, FOXA2 has been reported to inhibit tumorigenesis both in vitro and in vivo [59]. Additionally, FOXA2 and CDX2 cooperate with NKX2-1 to inhibit metastasis in lung adenocarcinoma [60]. In another study, FOXA2 has been reported to inhibit mesenchymal transition in breast cancer through E-cadherin and ZEB-1 regulation [24]. Additionally, it has been found that FOXA2 interacts with other proteins to inhibit the proliferation and migration of breast cancer cells [25,45]. However, FOXA2 mRNA has also been reported to be associated with relapse in basal-like breast carcinoma [61]. Here, the contradictory role of FOXA2 in breast cancer may be associated with the localization of FOXA2. Our current results showed that cytoplasmic FOXA2 expression was higher in basal-type breast cancer cells compared to luminal-type cells. However, further investigations are needed, as we used only one basal cell line in this study. In luminal-type cells, FOXA2 expression was higher in the nucleus. Therefore, the subcellular localization of FOXA2 may influence its oncogenic or tumor-suppressive effects. However, further investigations are required to prove this possibility. It has been reported that FOXA2 is phosphorylated by AKT at the threonine residue at position 156, which is found within FOXA2’s nuclear export signal domain. FOXA2 phosphorylation at this residue leads to FOXA2 nuclear export [62]. Additionally, acetylation and deacetylation compete to influence FOXA2’s transcriptional activity. Lys259 (lysine259) on FOXA2 is deacetylated by SIRT1 (silent mating type information regulation 2 homolog) deacetylase when insulin is present, which reduces the target gene’s expression and boosts export from the nuclei in hepatocytes [63]. Additionally, Foxa2 has a functional CRM1 (Chromosomal Maintenance 1, also known as Exportin 1)-dependent leucine-rich nuclear export site that is required for nuclear exclusion in response to insulin stimulation.

Furthermore, CD44 is known to regulate cell function through various signaling pathways, such as Rho GTPases, Ras-MAPK, and PI3K/AKT [5,6,7]. A previous study showed that insulin regulated FOXA2 localization through AKT [62]. In this study, we found that CD44 also regulates FOXA2 localization through the AKT pathway. AKT phosphorylation mediated by CD44 promotes its translocation to the nucleus. As a consequence, it can phosphorylate FOXA2, which leads to FOXA2 accumulation in the cytosol, thereby reducing E-Cadherin expression. This indeed will promote a mesenchymal phenotype, resulting in enhanced cancer cell migration and invasion abilities.

## 5. Conclusions

In conclusion, our study showed that CD44 is more highly expressed in metastatic lymph nodes compared to primary tumors. We also provide evidence that the overexpression of CD44 in breast cancer markedly promoted cell migration and invasion abilities, while the opposite effects were observed upon CD44 knockdown. We summarize that CD44 promotes cancer cell migration through the cytosolic localization of FOXA2 mediated by the AKT signaling pathway. This study provides further insights in designing future therapeutic strategies for breast cancer.

## Data Availability

The data present in this study are available on request.

## Supplementary Materials

The following supporting information can be downloaded at https://www.mdpi.com/article/10.3390/biomedicines10102488/s1: Figure S1: Basal-type breast cancer cells have a high expression of CD44. (a) Representative figure showing the gating strategy for flow cytometric data for CD44-positive cells in primary tumors and metastatic lymph nodes. (b) Table showing details of the flow cytometric analysis of CD44 expression in primary breast tumors and paired metastatic lymph nodes and, also, the patients’ ER, PR, and HER2 status. (c) Immunoblots of CD44 in basal-type cells (HS578T and MDA-MB-231) and three luminal-type cells (MCF7, T47D, and ZR75). (d) Knockdown efficiency of CD44 lentivirus in MDA-MB-231 cells. (e) Cell morphology of CD44 knockdown MDA-MB-231 and ZR75 cells and CD44-overexpressed ZR75 and MCF7 cells by using optical microscopy. Scale bar = 50 µm. (f) Cell proliferation of CD44 knockdown MDA-MB-231 and CD44-overexpressed MCF-7 cells. Figure S2: mRNA level of CD44 and FOXA2. (a) Quantitative RT-PCR of CD44 and FOXA2 in nontargeting control and CD44 knockdown cells (MDA-MB-231). (b) Quantitative RT-PCR of CD44 and FOXA2 in nontargeting control and CD44-overexpressed cells (MCF7). Figure S3: Protein expressions of EMT markers in CD44 and FOXA2 double knockdown and double overexpressed cells. (a) Western blot showing the protein expression of mesenchymal markers snail, slug, twist, vimentin, and ZEB1, respectively, in CD44 knockdown MDA-MB-231 cells. (b) Western blot showing FOXA2 knockdown efficiency and E-cadherin protein expressions in CD44 knockdown MDA-MB-231 cells. (c) Western blot showing protein expressions of mesenchymal markers snail, twist, and ZEB1, respectively, in CD44-overexpressed, FOXA2-overexpressed, and double overexpressed (both CD44 and FOXA2) MCF7 cells. Figure S4: Effects of doxorubicin on (a) CD44 knockdown ZR75 cells and (b) CD44-overexpressed MCF7 and ZR75 cells. Figure S5: AKT is the possible kinase through which CD44 regulates FOXA2 localization. (a) Prediction of CD44-regulated protein kinases that may be associated with FOXA2. (b) p-AKT expression in CD44 knockdown T47D cells. (c) Immunofluorescence staining for FOXA2 (green) and CD44 (red) in wortmannin (Wo) and SC79 (SC)-treated MDA-MB-231 cells. Scale bar = 50 µm. Table S1: shRNA target sequence. Table S2: List of antibodies.
