# Supplementary material for: CD44 Promotes Breast Cancer Metastasis through AKT-Mediated Downregulation of Nuclear FOXA2
Source: Biomedicines. 2022 Oct 5;10(10):2488. doi: 10.3390/biomedicines10102488 (PMC9599046; doi:10.3390/biomedicines10102488)
Supplement: Supplementary file 1 [file biomedicines-10-02488-s001.zip › biomedicines-1836766-supplementary.pdf]

(a)

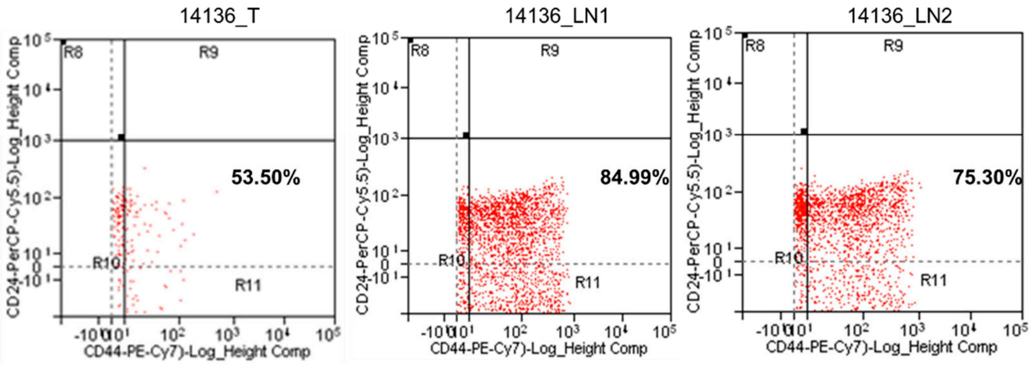

(b)

Patient\CSC Marker	CD44+	ER	PR	HER2	Histology
**14-136**	T(53.50%), LN1(84.99%), LN2(75.30%)	-	-	+	Infiltrating duct carcinoma
**14-173**	T(73.63%), ALN1(79.52%), ALN2(84.98%)	+	+	+	Infiltrating duct carcinoma
**14-174**	T(50.15%), LN1(97.27%), LN2(96.09%)	+	+	-	Mucinous adenocarcinoma
**14-215**	T(90.68%), LN1(82.79%), LN2(89.62%)	-	-	+	Infiltrating duct carcinoma
**14-241**	T(68.74%), LN1(90.90%), LN2(91.72%), LN3(89.16%)	-	-	+	Infiltrating duct carcinoma
**14-252**	T(54.90%), SLN1(91.93%), SLN2(90.97%)	-	-	+	Infiltrating duct carcinoma
**14-253**	T(37.37%), ALN(83.56%)	+	+	-	Infiltrating duct carcinoma
**14-374**	T(4.21%), ALN(5.58%)	-	-	-	Infiltrating duct carcinoma
**14-375**	T(34.50%), SLN(37.29%)	-	-	+	Infiltrating duct carcinoma
**14-377**	T(50.17%), ALN(72.14%)	+	-	+	Infiltrating duct carcinoma
**14-379**	T(33.19%), LN1(71.91%)	+	+	-	Infiltrating duct carcinoma
**14-413**	T(34.06%), SLN(1.90%)	-	-	+	Infiltrating duct carcinoma
**14-414**	T(9.15%), SLN(91.35%), ALN(88.02%)	-	-	-	Infiltrating duct carcinoma
**15-003**	T(10.11%), SLN(55.08%)	+	+	-	Infiltrating duct carcinoma

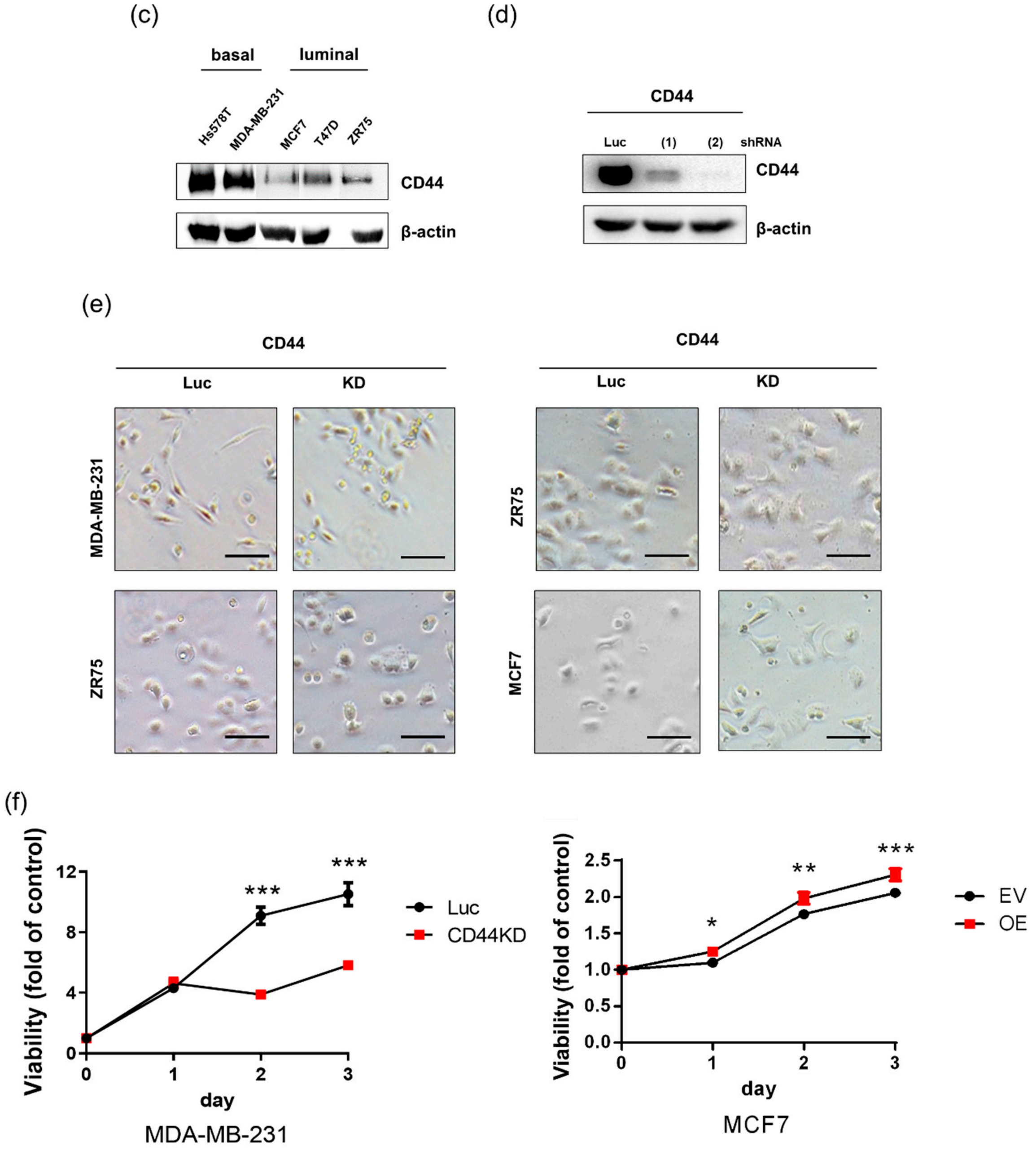

Figure S1: Basal type breast cancer cells have high expression of CD44. (**a**) Representative figure showing the gating strategy for flow cytometric data for CD44 positive cells in primary tumors and metastatic lymph nodes (**b**) Table showing details of flow cytometric analysis of CD44 expression in primary breast tumors and paired metastatic lymph nodes and also patients ER, PR and HER2 status. (**c**) Immunoblots of CD44 in basal type cells (HS578T and MDA-MB-231) and three luminal type cells (MCF7, T47D, and ZR75). (**d**) Knockdown efficiency of CD44 lentivirus in MDA-MB-231 cells. (**e**) Cell morphology of CD44 knockdown MDA-MB-231 and ZR75 cells and CD44 overexpressed ZR75 and MCF7 cells by using optical microscopy. Scale bar = 50μm (**f**) Cell proliferation of CD44 knockdown MDA-MB-231 and CD44 overexpressed MCF-7 cells.

(a)

Genes with upregulated mRNA level

Gene name	Fold change
BMI1	2.52
DLL1	2.26
DLL4	4.88
ERBB2	5.90
FOXA2	2.50
ITGA2	4.46
ITGA6	2.25
KLF4	2.32
MAML1	2.55
NOTCH1	3.60
PECAM1	2.80
POU5F1	5.68
PTCH1	3.00
TGFBR1	8.02
THY1	2.79
WEE1	3.79
YAP1	3.23
ZEB2	6.26

Genes with downregulated mRNA level

Gene name	Fold change
CD44	-7.12
DACH1	-3.02
EGF	-2.67
ENG	-16.18
EPCAM	-4.85

(b)

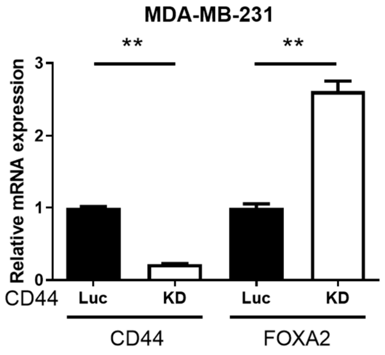

(c)

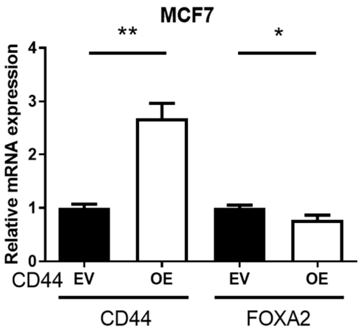

Figure S2: mRNA level of CD44 and FOXA2. (**a**) Genes with upregulated and downregulated mRNA level in CD44 knockdown MDA-MB-231 cells analyzed by RT2 array. (**b**) Quantitative RT-PCR of CD44 and FOXA2 in non-targeting control and CD44 knockdown cells (MDA-MB-231). (**c**) Quantitative RT-PCR of CD44 and FOXA2 in non-targeting control and CD44 overexpressed cells (MCF7).

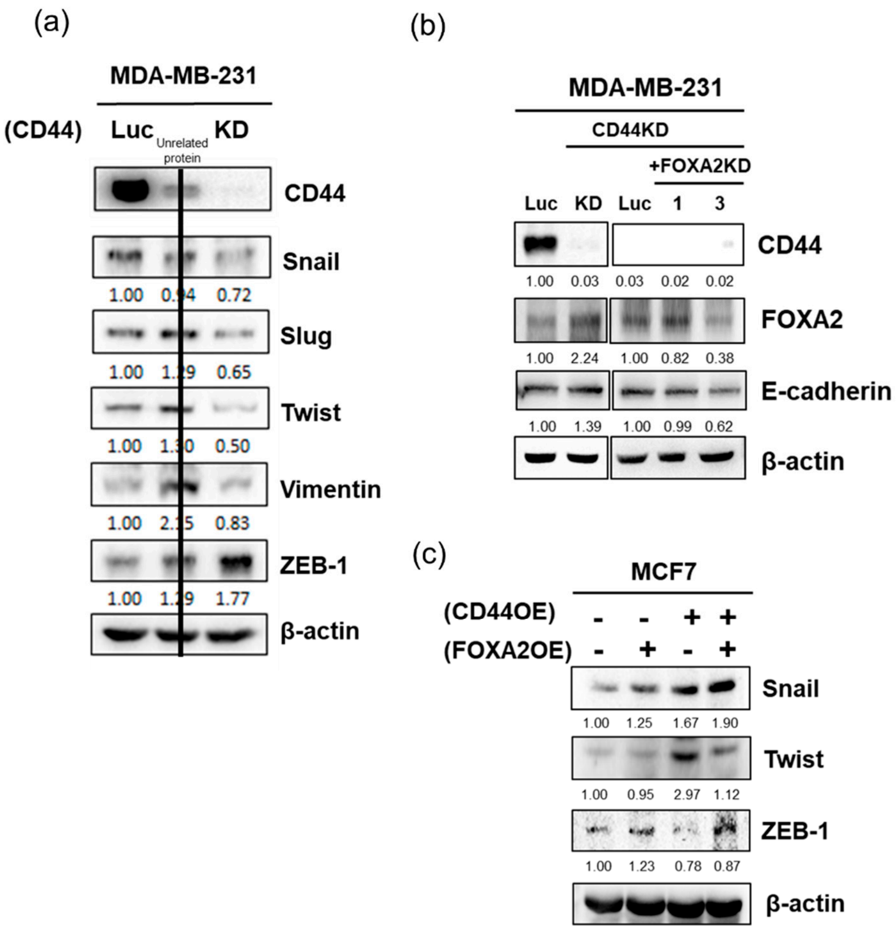

Figure S3: Protein expressions of EMT markers in CD44 and FOXA2 double knockdown and double overexpressed cells (**a**) Western blot showing protein expression of mesenchymal markers snail, slug, twist, vimentin, and ZEB1 respectively in CD44 knockdown MDA-MB-231 cells. (**b**) Western blot showing FOXA2 knockdown efficiency and E-cadherin protein expressions in CD44 knockdown MDA-MB-231 cells. (**c**) Western blot showing protein expressions of mesenchymal markers snail, twist, and ZEB1 respectively in CD44 overexpressed, FOXA2 overexpressed, and double overexpressed (both CD44 and FOXA2) MCF7 cells.

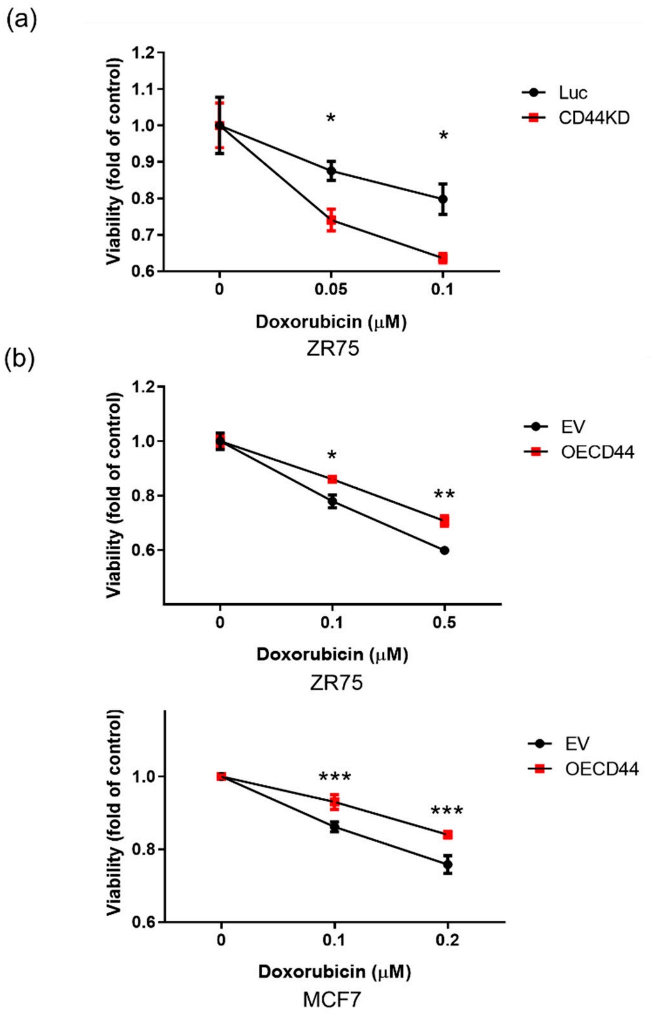

Figure S4.: Effects of doxorubicin on (**a**) CD44 knockdown ZR75 cells & (**b**) CD44 overexpressed MCF7 and ZR75 cells.

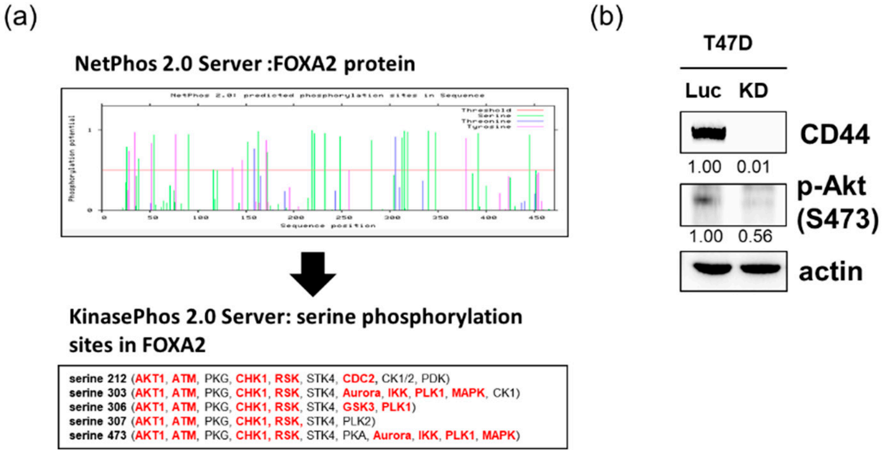

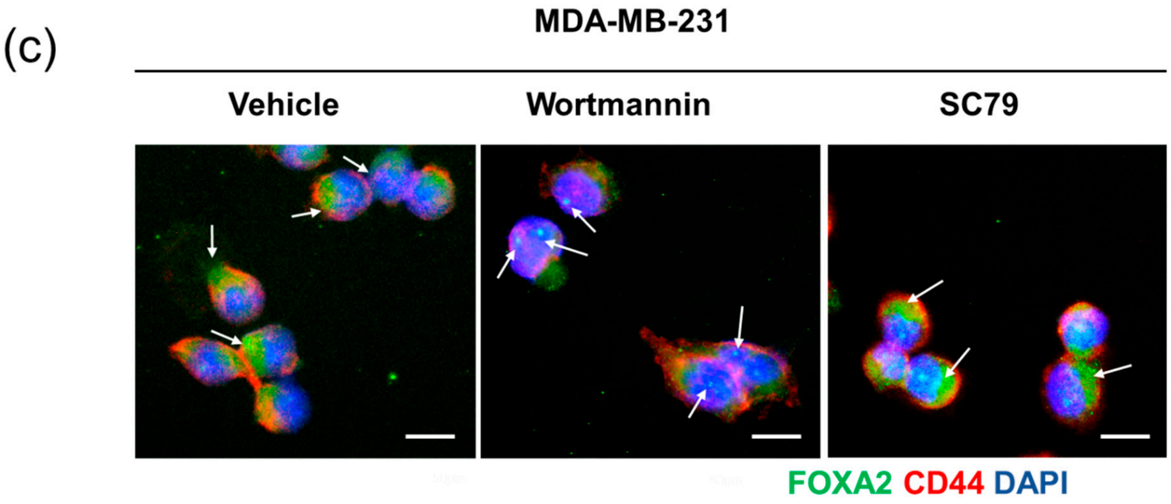

Figure S5: AKT is the possible kinase through which CD44 regulates FOXA2 localization. (**a**) Prediction of CD44-regulated protein kinases that may be associated with FOXA2. (**b**) p-AKT expression in CD44 knockdown T47D cells. (**c**) Immunofluorescence staining for FOXA2 (green) and CD44 (red) in wortmannin (Wo) and SC79 (SC) treated MDA-MB-231 cells. Scale bar = 50μm.

Table S1. shRNA target sequence

Gene symbol	Sequence	Region	Score
CD44_clone1 (pLKO_005)	GGACCAATTACCATAAC TATT	CDS	TRC,# TRCN0000296191
CD44_clone2 (pLKO_005)	CCGTTGGAAACATAAC CATTA	3UTR	TRC,# TRCN0000308110
FOXA2_clone1 (pLKO.1)	GCAAGGGAGAAGAAAT CCATA	3UTR	TRC,# TRCN0000014913
FOXA2_clone3 (pLKO.1)	GAACGGCATGAACACG TACAT	CDS	TRC,# TRCN0000014915
Shluc (Luciferase)	GCGGTTGCCAAGAGGT TCCAT	3UTR	TRC,# TRCN0000072249

Table S2. List of antibodies

Antibody	Catalog number	Dilution
Slug	PA5-20290	1.1000
Snail	GTX82509	1:1000
Twist	GTX127310	1:2000

Vimentin	GTX100619	1:3000
ZEB1	GTX105278	1: 2000
